# Advances and efficacy in specimen handling for endoscopic ultrasound‐guided fine needle aspiration and biopsy: A comprehensive review

**DOI:** 10.1002/deo2.350

**Published:** 2024-03-15

**Authors:** Takuji Iwashita, Shinya Uemura, Tezuka Ryuichi, Akihiko Senju, Shota Iwata, Yosuke Ohashi, Masahito Shimizu

**Affiliations:** ^1^ First Department of Internal Medicine Gifu University Hospital Gifu Japan

**Keywords:** deep learning, histological sampling, macroscopically visible core, stereomicroscopy on‐site evaluation, visual on‐site evaluation

## Abstract

Endoscopic ultrasound‐guided fine needle aspiration and biopsy have significantly evolved since they offer a minimally invasive approach for obtaining pathological specimens from lesions adjacent to or within the intestine. This paper reviews advancements in endoscopic ultrasound‐guided fine needle aspiration and biopsy techniques and devices, emphasizing the importance of handling specimens for diagnostic accuracy. Innovations of fine needle biopsy needles with features like side holes and Franseen shapes have enhanced histological sampling capabilities. Techniques for specimen handling, including rapid on‐site evaluation and macroscopic on‐site evaluation, play pivotal roles in assessing sample adequacy, thereby influencing diagnostic outcomes. The utility of artificial intelligence in augmenting rapid on‐site evaluation and macroscopic on‐site evaluation, although still in experimental stages, presents a promising avenue for improving procedural efficiency and diagnostic precision. The choice of specimen handling technique is dependent on various factors including endoscopist preference, procedure objectives, and available resources, underscoring the need for a comprehensive understanding of each method's characteristics to optimize diagnostic efficacy and procedural safety.

## BACKGROUND

Endoscopic ultrasound‐guided fine needle aspiration (EUS‐FNA) is a technique utilized for puncturing lesions adjacent to or within the intestine, employing real‐time EUS imaging guidance from the digestive tract to procure pathological specimens. Since its first documentation in 1992,^1^ EUS‐FNA has been widely adopted as a precise and minimally invasive approach in clinical settings.^2^, ^3^ The intervening decades have witnessed significant advancements in both the techniques and devices employed in EUS‐FNA, aimed at enhancing the efficiency of pathological specimen collection. Originally, the FNA needles were confined to Lancet or Menghini designs, with a limited needle gauge of 22 or 19, made from stainless steel. This was followed by the development of thinner needles, specifically 25‐gauge, fabricated from flexible materials such as nickel‐titanium to primarily improve maneuverability. Recent innovations have introduced fine needle biopsy (FNB) needles, distinguished by modifications to the needle tip—including side holes, reversed bevels, Franseen shapes, or fork‐tip designs—to augment the efficiency of pathological specimen sampling. The advent of FNB needles has transitioned the practice to be known as EUS‐FNB, denoting a significant advancement in histological sampling capabilities provided by these needles. Furthermore, enhancements in techniques during EUS‐FNA, such as variations in the degree of negative pressure or the puncture methodology, have been developed and evaluated by various researchers. The selection of a particular technique is often at the discretion of the endoscopist or the facility. While these advancements in devices and methodologies have undeniably improved sampling efficacy, the management of the pathological specimens obtained by EUS‐FNA is pivotal for sustaining and augmenting diagnostic accuracy and has been the focus of extensive research. This paper aims to review the current practices in the management of pathological specimens obtained by EUS‐FNA, underscoring its critical role in the diagnostic process.

### Handling of pathological specimen

Handling of pathological specimens obtained through EUS‐FNA is crucial for maximizing the procedure's efficacy and safety. Beyond standard cytologic and histologic analyses, various strategies have been employed to assess the quality of the FNA samples, proving instrumental in determining the appropriate timing to conclude the FNA procedure or in making decisions regarding the treatment strategy based on preliminary diagnoses. Rapid on‐site evaluation (ROSE) is a widely recognized method for assessing cytologic findings immediately, utilizing a simply stained smear slide within the procedure room. Additionally, macroscopic on‐site evaluation (MOSE) offers another approach, where the specimen obtained by FNA is examined through gross inspection, focusing on the presence of whitish material as an indicator of sample quality. In a more innovative vein, artificial intelligence (AI) has been explored as a means to enhance ROSE and MOSE by aiming for a more generalized application. Although the integration of AI in ROSE and MOSE is currently in the experimental stages, its potential to improve diagnostic accuracy and procedural efficiency represents a promising advancement in the field of gastrointestinal endoscopy.

### Rapid on‐site evaluation

In ROSE, a part of cytologic slides obtained by EUS‐FNA are subjected to a rapid staining method in the endoscopy suite. Diff‐Quik stain, a streamlined and expedited variant of the May–Grünwald–Giemsa stain, is the preferred staining technique for ROSE, necessitating only a few minutes to complete. Cytopathologists provide immediate feedback to the endoscopist regarding the cytologic findings from Diff‐Quik stained slides. This allows the endoscopist to determine the optimal timing to discontinue EUS‐FNA when adequate cytologic material is evident. Consequently, ROSE has the potential to decrease the number of FNA passes compared to the uniform execution of an optimal number of FNA passes.^4^, ^5^, ^6^ Moreover, ROSE enhances the rate of acquiring pathological material and improves diagnostic accuracy. Furthermore, it facilitates the preliminary determination of the treatment course based on ROSE findings, pending the final pathological outcome.

Several meta‐analyses have been conducted to evaluate ROSE during EUS‐FNA, reflecting the varied investigations into its utility. A meta‐analysis assessing the diagnostic efficacy of EUS‐FNA for pancreatic lesions included 15 studies with 1860 patients, revealing a pooled sensitivity of 92% (95% confidence interval [CI], 91%–93%) and specificity of 96% (95% CI, 93%–98%).^7^ Subgroup analysis differentiated between six studies with ROSE and nine studies without, indicating a pooled sensitivity of 95% (95% CI, 93%–96%) and 89% (95% CI, 86%–91%), respectively.^7^ Another meta‐analysis focused on ROSE's impact on the adequacy of EUS‐FNA for solid pancreatic lesions encompassed 70 studies, concluding that ROSE significantly improved the overall adequacy rate by up to 3.5%.^8^ These analyses suggest that incorporating ROSE into EUS‐FNA enhances sample adequacy for pathological evaluation and the diagnostic performance of EUS‐FNA. However, not all centers have access to a cytopathologist for ROSE. Two randomized controlled trials have compared ROSE conducted by endoscopists. One randomized controlled trial by Nevel et al.^9^ included 33 patients in the ROSE group and 32 patients in the non‐ROSE group, undergoing EUS‐FNA for solid pancreatic lesions. The ROSE group demonstrated a shorter procedural duration (30.0 vs. 37.0 min, *p* < 0.005) and fewer needle passes (2.6 vs. 3.5 passes, *p* < 0.005), although no differences in sample adequacy or diagnostic yield were reported. Another randomized controlled trial by Zhang et al.^10^ compared ROSE performed by endoscopists in 97 patients to 97 patients without ROSE, also targeting solid pancreatic lesions. This study reported higher accuracy (94.8% vs. 70.1%, *p* < 0.001) and sensitivity (94.4% vs. 65.1%, *p* < 0.001) in the ROSE group, yet no differences were found in the adequacy rate or number of punctures. Overall, ROSE, whether performed by a cytopathologist or an endoscopist, appears to enhance diagnostic capabilities and increase the efficiency of EUS‐FNA, necessitating fewer passes and reducing procedure time.

The application of EUS‐FNB has enhanced sampling and diagnostic capabilities. A prospective pilot study assessing the diagnostic yield of EUS‐FNB with three passes for solid pancreatic tumors demonstrated a high diagnostic accuracy of 96% (72/75), with a plateau in diagnostic yield after two passes of FNB.^11^ A comprehensive retrospective study involving 3020 patients compared EUS‐FNA (68.9%) and FNB (31.1%), revealing that the median number of passes for diagnostic adequacy with ROSE was significantly fewer for FNB than for FNA (1 [interquartile range {IQR}: 1–2] vs. 2 [IQR: 1–3], *p* < 0.001). Moreover, the diagnostic yield on cell block was significantly better with FNB (92.3% versus 71.1%, *p* < 0.001).^12^ The superior sampling and diagnostic performance of EUS‐FNB may be leading to a reduced need for ROSE. A recent randomized controlled trial that compared EUS‐FNB with ROSE (385 patients) and without ROSE (386 patients), where three FNB passes were performed for solid pancreatic lesions, showed similar diagnostic accuracies (96.4% with ROSE and 97.4% without ROSE, *p* = 0.396), confirming the noninferiority of EUS‐FNB without ROSE.^13^ Additionally, a significantly higher tissue core rate was achieved by EUS‐FNB without ROSE (70.7% versus 78.0%, *p* = 0.021), with a significantly reduced mean sampling procedural time (17.9 vs. 11.7 min, *p* < 0.0001); however, the safety and histologic sample quality was comparable. While the reliance on ROSE has decreased with EUS‐FNB, it still offers preliminary diagnoses during procedures and may reduce the number of required passes. Further studies are warranted to evaluate the utility of ROSE in conjunction with EUS‐FNB.

### Macroscopic on‐site evaluation

**FIGURE 1:**
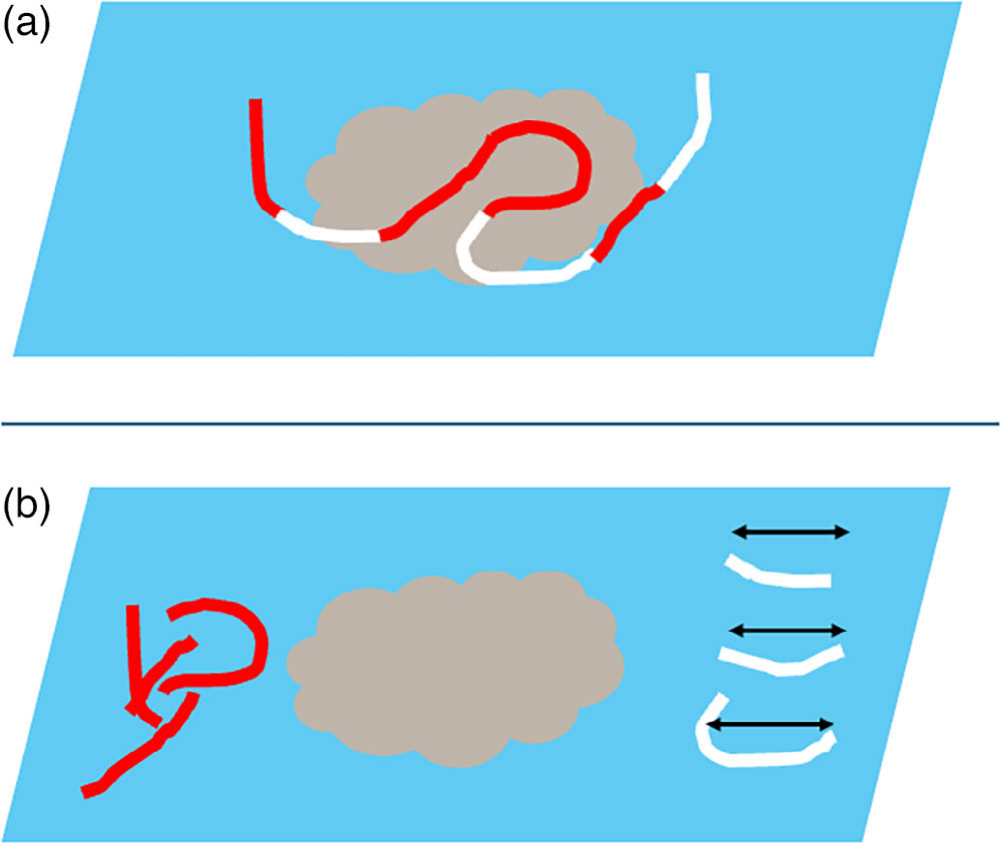
The flow of macroscopic on‐site evaluation. (a) The specimen obtained by endoscopic ultrasound‐guided fine needle aspiration or biopsy is expelled on a glass slide. (b) Whitish macroscopic visible cores are separated on the slide and the length of the cores is measured.

ROSE is not universally accessible across various centers due to constraints in human and financial resources, despite extensive research validating its effectiveness in EUS‐FNA procedures. One proposed solution to this limitation is the implementation of ROSE by endoscopists, although this may augment the endoscopists' workload. Consequently, there has been a call for a simpler and more efficient technique for assessing the quality of FNA samples for suitability in pathological analysis. MOSE, introduced by Iwashita et al.,^14^ presents a direct method enabling endoscopists to evaluate the quantity of pathological specimens. Within MOSE, the FNA sample is dispensed onto a glass slide after reinserting the needle stylet, and the presence of a macroscopically visible core (MVC) — a discernible whitish or yellowish tissue fragment — is examined (Figure 1). Findings indicate that an MVC measuring ≥ 4 mm reliably predicts the presence of a histologic core, with significantly greater sensitivity in histologic (92.4% vs. 40.8%, *p* < 0.0001), cytologic (74.2% vs. 34.7%, *p* < 0.0001), and overall (95.5% vs. 57.1%, *p* < 0.0001) evaluations when utilizing a 19‐gauge FNA needle during EUS‐FNA. Subsequently, Chong et al.^15^ conducted a randomized controlled trial to compare EUS‐FNA using a 19‐gauge FNA needle with MOSE against a conventional method, which involved three to five passes, for extraintestinal solid lesions sized ≥ 2 cm. The trial defined completion of EUS‐FNA in the MOSE group upon acquisition of an MVC — identified as whitish or yellowish tissue fragments with substantial bulk exceeding 4 mm. The study, including 244 patients (122 in the conventional group and 122 in the MOSE group), demonstrated a significant reduction in the number of FNA passes required in the MOSE group (median number of passes: 2 in MOSE vs. 3 in conventional; *p* < 0.001) while maintaining comparable diagnostic yields (92.6% in MOSE vs. 89.3% in conventional; *p* = 0.37). These results position MOSE as a practical alternative for obtaining adequate tissue samples in settings where ROSE is not available, particularly during EUS‐FNA with a 19‐gauge FNA needle for solid masses.

The utility of MOSE in EUS‐FNA has been established, and its effectiveness during EUS‐FNB has also been assessed recently. A meta‐analysis examining the diagnostic parameters of EUS‐guided tissue acquisition with MOSE included 14 studies with 1508 lesions in 1489 patients.^16^ It revealed that the pooled accuracy of FNA and/or FNB specimens for achieving a pathologic diagnosis via MOSE was 91.3%, with a pooled sensitivity of 91.5% and a pooled specificity of 98.9%. Subgroup analyses utilizing newer‐generation FNB needles, such as those with Franseen or Fork‐tip shapes, demonstrated similar pooled rates of accuracy, sensitivity, and specificity at 90.6%, 91.5%, and 98.2%, respectively. The study determined that MOSE exhibited excellent diagnostic parameters for both EUS‐FNA and FNB. In a more recent study, Mangiavillano et al.^17^ performed a multicenter randomized controlled trial comparing EUS‐FNB using a 22‐gauge Franseen needle for solid masses with MOSE to EUS‐FNB without MOSE. The MOSE arm ceased puncture if MOSE identified whitish or yellowish worm‐like cores exceeding 10mm in length. Conversely, the non‐MOSE EUS‐FNA protocol involved three needle passes per lesion. Involving 370 patients (190 in the MOSE group and 180 in the non‐MOSE group), the trial found no significant differences in diagnostic accuracy (90.0% for MOSE vs. 87.8% for non‐MOSE, *p* = 0.49), sample adequacy (93.1% for MOSE vs. 95.5% for non‐MOSE, *p* = 0.31), or adverse event rates (2.6% for MOSE vs. 1.1% for non‐MOSE, *p* = 0.28). However, the median number of passes was significantly lower in the MOSE group (1 vs. 3 passes; *p* < 0.001). The study concluded that MOSE effectively determines sample adequacy and reduces the number of needle passes necessary for diagnosis during EUS‐FNB with a 22‐gauge FNB needle. These findings suggest that MOSE is a valuable tool for estimating the number of specimens suitable for histological evaluation in EUS‐FNB of solid tumors, although the optimal cut‐off length of MVC should be ascertained for each needle size.

Several methods for assessing specimens collected by EUS‐FNA or EUS‐FNB have been described. This includes MOSE, where the specimen is visually inspected on a glass slide with the unaided eye. In visual on‐site evaluation (VOSE), specimens from EUS‐FNB using either 22‐ or 25‐gauge Franseen needles are fully expelled into a graduated formalin vial through reinsertion of the stylet.^18^ The vial is then sealed and gently shaken to aid in the visual estimation of the MVC by the naked eye. This study assessed MVC presence based on the descriptors “single or multiple,” “long (>4 mm) or short (≤4 mm),” and “white or red‐mixed.” Findings indicate that “red‐mixed specimens” correlate with a higher probability of histological adequacy (odds ratio 2.39), suggesting their predictive value for histological sufficiency. Additionally, stereomicroscopy on‐site evaluation (SOSE) has been implemented, wherein specimens collected by EUS‐FNA with 22‐gauge needles are placed in a Petri dish and scrutinized under a stereomicroscope at 30x magnification to identify a white‐colored core.^19^ The sensitivity for identifying adequate specimens was 91.4% with a core length cutoff of ≥ 11 mm, and multivariate analysis substantiated that core length in SOSE is an important determinant for tissue diagnosis. These on‐site evaluation methods for EUS‐FNA or EUS‐FNB specimens may also be useful in determining histological adequacy.

### Artificial intelligence

The landscape of AI within the medical domain is undergoing rapid evolution, markedly influencing various facets of healthcare delivery. AI technologies are being employed to augment diagnostic processes, disease management, and the optimization of healthcare operations, among other uses. Specifically, the application of AI in EUS, EUS‐FNA, and EUS‐FNB has primarily been investigated in the context of differential diagnoses or analyses based on EUS imagery.^20^, ^21^, ^22^, ^23^ However, the potential of AI in the processing of pathological specimens obtained by EUS‐FNA remains largely unexplored. In the study conducted by Lin et al.^24^ evaluating AI‐assisted ROSE, a total of 467 digitized images of Diff‐Quik stained EUS‐FNA slides were segmented into training (3642 tiles from 367 images) and internal validation sets (916 tiles from 100 images). Each tile was categorized as either positive or negative for cancer cells. Deep learning was executed utilizing a model developed with Keras (TensorFlow), incorporating ResNet101V2 as its architecture. The efficacy of AI‐assisted ROSE in distinguishing positive from negative cancer cell samples demonstrated sensitivity, specificity, and accuracy rates of 96.0%, 98.5%, and 97.7% in the training set, and 79.1%, 85.4%, and 83.4% in the internal validation set, respectively. Further, Ishikawa et al.^25^ assessed the performance of AI‐based MOSE on specimens collected via EUS‐FNB using a 22‐gauge Franseen needle for pancreatic tumors. Specimens were expelled onto a Petri dish with saline, and images of the solid components were captured using a stereomicroscope. Deep learning, employing an image recognition neural network (AlexNet), was then applied to predict the presence of diagnosable material for histology based on stereomicroscopic images of 98 specimens. Of these, 81 were deemed histologically diagnosable, and 17 were not. The diagnostic performance of AI‐based MOSE did not surpass that of expert EUS evaluations, showing a sensitivity of 85.8% versus 88.9%, a specificity of 55.2% versus 47.1%, and an accuracy of 71.8% versus 81.6%. However, the inclusion of histologic images in the evaluation enhanced the performance metrics of AI‐based MOSE. Given the rapid advancement and adoption of AI in medical fields, AI‐assisted ROSE and MOSE could potentially be integrated into clinical practice shortly. Nevertheless, the application of AI in the analysis of EUS‐FNA specimens remains within the research phase at present.

## CONCLUSION

The handling of specimens retrieved via EUS‐FNA or EUS‐FNB is crucial for optimizing the effectiveness and safety of the procedure. A summary of each handling method is presented in Table 1. Predicting the quantity and quality of samples with either method is deemed beneficial for minimizing the number of punctures and avoiding sample inadequacy. However, the optimal handling method may vary based on the endoscopist's preference, the objectives of EUS‐FNA, the facility's circumstances, or the needles utilized. In recent years, obtaining tissue specimens for cancer genomic profiling has been added as a new role of EUS‐guided tissue acquisition.^26^, ^27^, ^28^ Therefore, the selection of specimen processing methods should be chosen with a thorough understanding of the characteristics of each method.

**TABLE 1:** Comparison of evaluation methods.

Method	Preparation	Evaluation	Merit	Demerit
ROSE	Diff‐Quik stained cytological slide	Microscopic evaluation by a cytology specialist	Adequacy of the specimen evaluated Preliminary diagnosis possible	Specialist required
MOSE	Specimens on a slide glass	Gross inspection by an endoscopist	No specialist needed for the evaluation Estimation of specimen amount Quick and simple	No quality information No preliminary diagnosis
VOSE	Specimens in formalin vial	Gross inspection by an endoscopist	No specialist needed for the evaluation Estimation of specimen amount Quick and simple	No quality information No preliminary diagnosis
SOSE	Specimens in Petri dish	Stereoscopic evaluation by an endoscopist	No specialist needed for the evaluation Estimation of specimen amount	No quality information No preliminary diagnosis Stereoscopy required
AI	As per ROSE or MOSE	Evaluation of images by AI	No specialist needed for the evaluation Facilitates quality control	Research protocol

Abbreviations: AI, artificial intelligence; MOSE, macroscopic on‐site evaluation; ROSE, rapid on‐site evaluation; SOSE, stereomicroscopy on‐site evaluation; VOSE, visual on‐site evaluation.

## CONFLICT OF INTEREST STATEMENT

None.
